# Kinetics and Quality of Microwave-Assisted Drying of Mango (*Mangifera indica*)

**DOI:** 10.1155/2016/2037029

**Published:** 2016-01-03

**Authors:** Ernest Ekow Abano

**Affiliations:** Department of Agricultural Engineering, School of Agriculture, College of Agricultural and Natural Sciences, University of Cape Coast, Cape Coast, Ghana

## Abstract

The effect of microwave-assisted convective air-drying on the drying kinetics and quality of mango was evaluated. Both microwave power and pretreatment time were significant factors but the effect of power was more profound. Increase in microwave power and pretreatment time had a positive effect on drying time. The nonenzymatic browning index of the fresh samples increased from 0.29 to 0.60 while the ascorbic acid content decreased with increase in microwave power and time from 3.84 mg/100g to 1.67 mg/100g. The effective moisture diffusivity varied from 1.45 × 10^−9^ to 2.13 × 10^−9^ m^2^/s for microwave power range of 300-600 W for 2 to 4 minutes of pretreatment. The Arrhenius type power-dependent activation energy was found to be in the range of 8.58–17.48 W/mm. The fitting of commonly used drying models to the drying data showed the Midilli et al. model as the best. Microwave power of 300 W and pretreatment time of 4 minutes emerged as the optimum conditions prior to air-drying at 7°C. At this ideal condition, the energy savings as a result of microwave application was approximately 30%. Therefore, microwave-assisted drying should be considered for improved heat and mass transfer processes during drying to produce dried mangoes with better quality.

## 1. Introduction

Mango (*Mangifera indica* L.) is one of the most popular fruits among millions of people in many countries worldwide. In Ghana, mango is grown in savannah and transitional areas by smallholder farmers and continues to remain a seasonal crop. Statistics point to annual production of 95,460 tonnes in 2013 [1]. In the diet of humans, mango plays important role; it provides the diet with colour, phytochemicals, and nutrients. The average mango composition is water (83 g/100 g), carbohydrate (15.2 g/100 g), sugar (13.7 g/100 g), fibre (1.8 g/100 g), fats (0.38 g/100 g), proteins (0.510 g/100 g), vitamins (mainly vitamin A, 389 mg/100 g, and vitamin C, 36 mg/100 g), and minerals (mainly potassium 168 mg/100 g and phosphorous 14 mg/100 g) [2]. The fruit is an excellent source of antioxidants including ascorbic acid. It provides about 50% of the recommended daily intake of vitamin C [3] and contains high amounts of beta-carotene, which is responsible for the typical yellow colour of the mangoes. Beta-carotene is very beneficial for humans as it is a provitamin A and antioxidant [4]. The pulp is found to contain pigment carotenoids, polyphenols, and omega-3 and omega-6 polyunsaturated fatty acids [5]. Fresh ripe mango contains more than 80 g/100 g water within a soft-pulpy cell wall structure, which is responsible for the fast decay after harvest. Therefore, the right postharvest processing intervention is required to prolong the shelf-life of mangoes.

Drying is among the methods for the purpose to produce high quality dried products, which can be consumed directly or used as ingredient for the preparation of chutneys, cakes, muesli, and oat granola. Conventional air-drying has been widely used in industrial drying of food products but this method is energy-intensive and time-consuming and often produces poor quality products. Many authors have reported that this method leads to degradation of products flavour, colour, nutrients, and case hardening, due to their long drying times and high temperatures employed in practice [6, 7]. Hence, combination of advanced drying methods with the conventional hot air is often recommended to reduce long drying times and poor product quality associated with conventional hot air-drying. Combination of osmotic dehydration preceded by microwave-assisted hot air-drying of mango has been studied [8]. The effect of gluten coating on osmotic dehydration of mango cubes has similarly been investigated [9]. Application of microwave prior to conventional drying of mango (*Mangifera indica* L.) is uncommon in scientific literature.

Microwave offers advantages that have been employed prior to or with conventional drying in food processing technologies. Several researchers have provided strong evidence that microwave-assisted drying is ideal for fruits and vegetables [10–14], which speed up drying process, increase mass transfer, and produce good quality products. Therefore, in this present work, the effect of microwave power and time as pretreatment to convective air-drying of mango slices was investigated.

## 2. Materials and Method

### 2.1. Sample Preparation

Fresh Kent mango fruits were obtained from the Abura Market, Cape Coast, Ghana. Selection of mango samples was based on visual assessment of uniform colour and geometry. The mangoes were washed under running tap water, peeled, and sliced into sizes of 50 mm × 25 mm × 10 mm using a stainless steel knife and immediately kept at −24°C to slow down physiological and chemical changes. Prior to the test, the samples were allowed to warm up to room temperature conditions. The initial moisture content of the mangoes was obtained from drying 5 g samples in an oven at 105°C for 24 hours according to the AOAC (1990) method. The initial total soluble solids were determined to be 16.7% with a refractometer (ABBE 98 490, Holland).

### 2.2. Experimental Design

A two-factor, 3-level factorial design was used for the experiment. The effect of two independent variables, microwave power *X*_1_ (300–600 W) and pretreatment time *X*_2_ (2–4 min), on three response variables, drying time, ascorbic acid, and nonenzymatic browning, was evaluated (Table 2).

### 2.3. Microwave Pretreatment

A domestic Samsung microwave machine with varied powers was used to predry 100 g of mango slices at power intensities of 300 W, 450 W, and 600 W for 2, 3, and 4 minutes prior to convective hot air-drying. After the microwave pretreatment, the slices were removed, weighed, and immediately subjected to convective air-drying. 100 g of mango slices without microwave pretreatment was used to serve as the control.

### 2.4. Drying Equipment and Drying Procedure

Microwave pretreated samples were transferred to a hot air cabinet dryer (GENLAB Oven, Model SDO225, 240 AC 1PH, 540 × 920 × 440 mm, 2 kW) set at temperature of 70°C and air circulation of 0.5 m/s. This temperature was chosen based on previous optimization studies [14]. The dryer was run idle for 1 hr earlier to the drying experiment. During drying, the masses of the samples were monitored every 30 min at the initial stages and later changed to 1 hr at the later stages of drying until constant mass was reached by a digital balance with an accuracy of ±0.001 g. For measuring the weight of the sample during experimentation, the tray with sample was taken out of the drying chamber, weighed on the digital top pan balance, and placed back into the chamber within 10 seconds.

### 2.5. Determination of Ascorbic Acid (Vitamin C)

Ascorbic acid content of the samples before and after drying was assayed colorimetrically following Roe and Kuether [19]. Two grams of dried mango slices was ground finely using a mortar and a pestle and placed in a 25 mL volumetric flask with 4% oxalic acid solution. The mixture was centrifuged and 10 mL of the supernatant was transferred into a conical flask after which bromine water was added in drops with constant mixing until the extract turns orange yellow. The solution was made up to 25 mL with 4% oxalic acid solution. Similarly, 10 mL of the stock ascorbic acid solution was converted into dehydroform by bromination. Again, 10 mL of standard dehydroascorbic acid solution was pipetted into a series of tubes. Aliquot (2 mL) of brominated sample extract was similarly pipetted out differently. The volume in each tube was made up to 3 mL by adding distilled water. One millilitre of DNPH (2,4-dinitrophenylhydrazine) reagent was added, followed by 1-2 drops of thiourea to each tube. A blank was set with water instead of ascorbic acid solution. The content of the tubes was placed on a shaker to mix and incubated at 37°C for 3 hours in a water bath. After incubation, the orange red osazone crystals formed were dissolved by adding 7 mL of 80% sulphuric acid. A graph of ascorbic acid concentration versus absorbance at 540 nm was plotted (*R* ^2^ = 0.9973) and used to calculate the ascorbic acid content in the sample.

### 2.6. Nonenzymatic Browning Determination

A method previously reported by [14] was used to evaluate the nonenzymatic browning of the dried mango slices. The extent of browning was measured as absorbance at 440 nm. Brown pigment formed was extracted from the test portions of the dried mango slices. Two-gram sample was ground into fine powder, after which 50 mL of ethanol (60%, v/v) was added and allowed to stand for 12 hours. The mixture was stirred slowly and filtered through 0.45 *μ*m nylon filter membrane. Browning index of filtrates was estimated by a spectrophotometer against 60% ethanol as blank. All samples were extracted in triplicate.

### 2.7. Drying Kinetics

The drying kinetics of mango slices were expressed in terms of empirical models, where the experimental data obtained were plotted in the form of a dimensionless moisture ratio (MR) against drying time in minutes. The MR of the mango slices was determined using

(1) $$\begin{array}{c} MR=\frac{M-M_{e}}{M_{o}-M_{e}}, \end{array}$$

where MR is the moisture ratio, *M*_*o*_ is the initial moisture content (g water/g dry matter), *M* is the moisture content at any time (g water/g dry matter), and *M*_*e*_ is the equilibrium moisture content (g water/g dry matter) [20].

Three empirical drying models widely used in scientific literature, Page, Henderson and Pabis, and Logarithmic, were fitted to the experimental data set (MR, *t*) shown in Table 1 to describe the drying kinetics of mango slices. A nonlinear regression procedure of SPSS 20.0 [21] was used to determine the drying rate constant, *k*, and coefficients (*a*, *c*, *n*) in the empirical models. The modelling was characterized by the reduced chi-square (*χ* ^2^), root mean square error (RMSE), and the determination coefficient (*R* ^2^) [22] displayed in (2), (3), and (4), respectively. Consider

(2) χ2=∑i=1NMRexp,i−MRpred,i²N−z,

(3) RMSE=1N∑i=1NMRexp,i−MRpred,i²,

(4) R2=1−∑i=1NMRpred,i−MRexp,i2∑i=1NMRpred,i−MRpred,i2,

where MR_exp,*i*_ and MR_pred,*i*_ are the experimental and predicted moisture ratio, respectively, *N* is the number of observations, and *z* is the number of constants in the drying model.

### 2.8. Determination of Moisture Diffusivity

Fick's second law of diffusion, which characterizes moisture migration during thin layer drying of food materials, was used to calculate the effective moisture diffusivity, considering a constant moisture diffusivity, infinite slab geometry, and uniform initial moisture distribution [23]:

(5) $$\begin{array}{c} MR=\frac{8}{\pi^{2}}\overset{\infty }{\underset{n=0}{\sum }}\frac{1}{\left(2n+1\right)}\exp\left(\;-\frac{\left(2n+1\right)\pi^{2}}{{4L}^{2}}D_{eff}t\;\right), \end{array}$$

where *D*_eff_ is the effective moisture diffusivity (m^2^/s) and *L* is half the thickness of slice of the sample (m). Equation (5) can be simplified to the following for long drying times:

(6) $$\begin{array}{c} MR=\frac{8}{\pi^{2}}\exp\left(\;-\frac{\pi^{2}D_{eff}t}{4L^{2}}\;\right). \end{array}$$

*D*_eff_ of the mango slices was obtained from the slope (*K*) of the graph of ln⁡MR against the drying time. ln⁡MR versus drying time (*t*) results in a straight line with a negative slope and *K* is related to *D*_eff_ by

(7) $$\begin{array}{c} K=\frac{\pi^{2}D_{eff}t}{4L^{2}}. \end{array}$$

### 2.9. Calculation of Activation Energy

According to Pillai et al. [24], for the standard microwave oven drying procedure, the internal temperature of sample is not an assessable variable. Therefore, the use of Arrhenius-type equation is considered for illustrating the relationship between the diffusivity coefficient and the ratio of the microwave power output to sample thickness instead of temperature for the calculation of the activation energy. The activation energy is found as modified from the revised Arrhenius. The equation as suggested by [25] is represented as

(8) $$\begin{array}{c} D_{eff}=D_{o}\exp\left[\;\frac{E_{a}q}{P}\;\right], \end{array}$$

where *D*_*o*_ is the constant in the Arrhenius equation (m^2^/s), *E*_*a*_ is the activation energy (W/mm), *P* is the microwave power (W), and *q* is the sample thickness (mm). Equation (8) can be rearranged as

(9) $$\begin{array}{c} \ln\left(\;D_{eff}\;\right)=\ln\left(\;D_{o}\;\right)-\frac{E_{a}q}{P}. \end{array}$$

The activation energy for moisture diffusion was obtained from the graph of ln⁡(*D*_eff_) against *q*/*P*.

### 2.10. Energy Consumption during Drying

The energy consumption in kWh of the microwave and the hot air dryer was calculated using

(10) $$\begin{array}{c} E_{c}=\frac{P\times t}{1000}, \end{array}$$

where *E*_*c*_ is the energy consumption in kWh, *P* is the power rating of either the microwave equipment or the convective air dryer in W, and *t* is the drying time in hours.

### 2.11. Optimization of the Drying Process

The optimization of the drying process was performed using a multivariate response method [26] with

(11) $$\begin{array}{c} DI={\left[\;\prod_{i=1}^{3}di\left(\;Y_{i}\;\right)\;\right]}^{1/3}. \end{array}$$

*di* represents the desirability for the various responses: drying time, ascorbic acid content, and nonenzymatic browning (*Y*_*i*_). The DI ranges between 0 and 1. Zero is the least preferred value while 1 is the most desired. Maximizing DI is the goal of optimization analysis. The optimization process incorporates goals and priorities for the factors and the responses. For this present study, the goal for the factors was at any level within the range of the design values, but, in the case of the responses, minimum values of drying time (DT), nonenzymatic browning index (BI), and maximum values of ascorbic acid (AA) were desired.

### 2.12. Statistical Analysis

A quadratic model was fitted to the average values of the responses to get the regression equations with design expert software [27]. The statistical significance of the model term was evaluated at 95% probability. The 3D plots for the factors were generated for the various responses. The accuracy of the model to describe the response variables was diagnosed by the determination coefficients (*R* ^2^) values and the nonsignificance of the lack of fit test.

## 3. Results and Discussion

### 3.1. Effect of Microwave Power and Pretreatment Time on Drying Kinetics of Mango Slices

**Figure 1:**
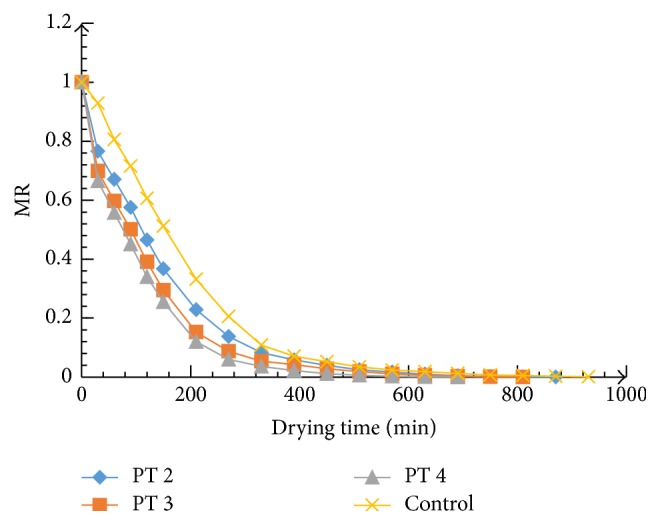
Variation of MR versus drying time at microwave power 300 W.

**Figure 2:**
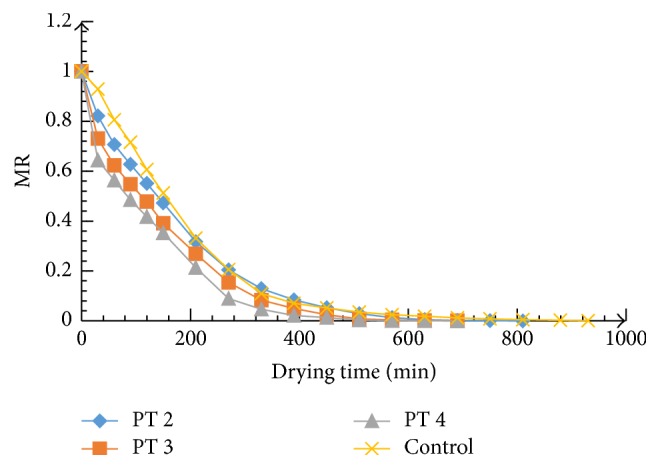
Variation of MR versus drying time at microwave power 450 W.

**Figure 3:**
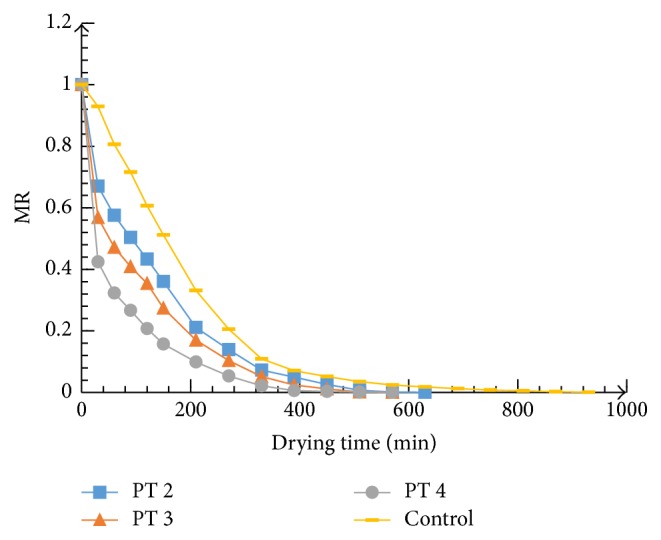
Variation of MR versus drying time at microwave power 600 W.

**Figure 4:**
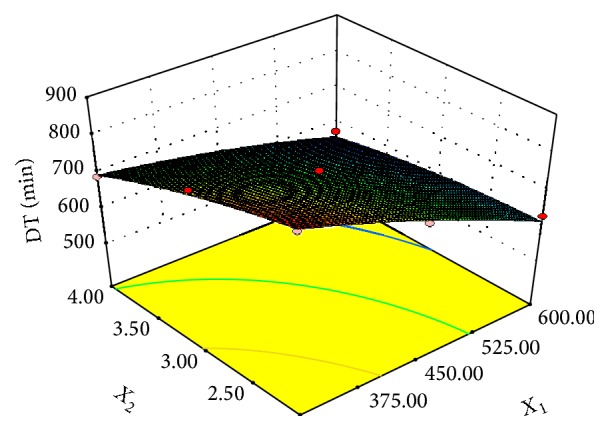
Effect of microwave power (*X*_1_) and microwave time (*X*_2_) on drying time.

Microwave power and pretreatment time increment had a positive effect on drying time for the 70°C dried samples as shown in Figures 1, 2, and 3. The initial average moisture content of the mangoes (*Mangifera indica*) was found to be 4.65 g moisture/g dry matter, which decreased to 0.03 g moisture/g dry matter (d.b.) after drying. The drying process generally followed a falling rate regime and the increase in microwave power and pretreatment time significantly (*P* ≤ 0.05) accelerated the drying process and increased energy efficiency. The estimated effect for each factor and the interaction between the variables were estimated (Table 3). Variation in the estimated coefficients shows that there were different contributions of the factors to drying time. Microwave power contributed 1.67 times higher than pretreatment time. As microwave power and pretreatment time increased, more moisture was removed and in the end resulted in the reduction in drying time (Figure 4). Drying time reduced from 870 min to 570 min as the microwave power and pretreatment time increased from 300 to 600 W and 2 to 5 min, respectively. This means that there was significant savings in time as microwave power pretreatment time increased.

The results corroborate what was reported by Rayaguru and Routray [28], Bai-Ngew et al. [29], and Karaaslan and Tunçer [30] for microwave-drying of* Pandanus amaryllifolius* leaves, durian chips, and spinach, respectively. Formation of porous structure in the tissues of mango as a result of electromagnetic waves application has been noted to be the plausible reason for the accelerated drying with microwave. In comparison with the control, microwave pretreatment enhanced heat and mass transfer within the mango tissues resulting in increased drying rates and energy utilization. The decrease in drying time with an increase in the microwave power density has been reported for other food materials, including tomato pomace [31], onions [32], apple pomace [33], and potatoes slices [34].

### 3.2. Effect of Microwave Power and Pretreatment Time on Moisture Diffusivity

**Figure 5:**
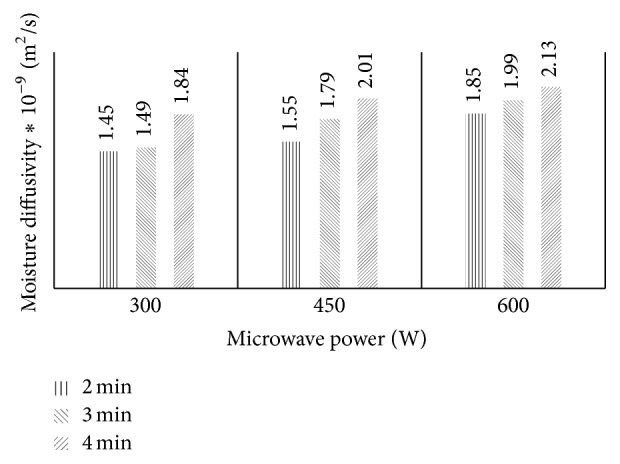
Effect of microwave power and time on moisture diffusivity of mango slices.

The variation of ln⁡(MR) against drying time plot for the various microwave power and pretreatment time used to calculate the various effective moisture diffusivity, *D*_eff_, had determination coefficient greater than 0.98. The effect on microwave power and pretreatment time on *D*_eff_ is evident (Figure 5). Effective moisture diffusivity coefficient increased with microwave power and pretreatment time. At a microwave power of 300 W, the *D*_eff_ values increased from 1.45 × 10^−9^ m^2^s^−1^ to 1.84 × 10^−9^ m^2^s^−1^ for samples pretreated for 2 to 4 min and dried at 70°C. A similar increase was observed for the *D*_eff_ values of the various microwave pretreatment conditions (Figure 5). The *D*_eff_ values obtained for microwave-assisted drying of mango slices lie within the general range of 10^−12^–10^−8^ m^2^s^−1^ for drying of food materials [17]. Increased heat energy as a result of increase in microwave power is reported to enhance the activity of the water molecules leading to higher moisture diffusivity [35]. Microstructure observation with scanning electron microscope (SEM) for microwave-assisted drying revealed creation of microchannel on the surface of the test samples. This shows that the higher the microwave power is and the longer the samples were treated, the higher the formation of porous structure is within the tissue of the mango slices to enhance heat and mass transfer, leading to higher moisture diffusivity rates.

**Figure 6:**
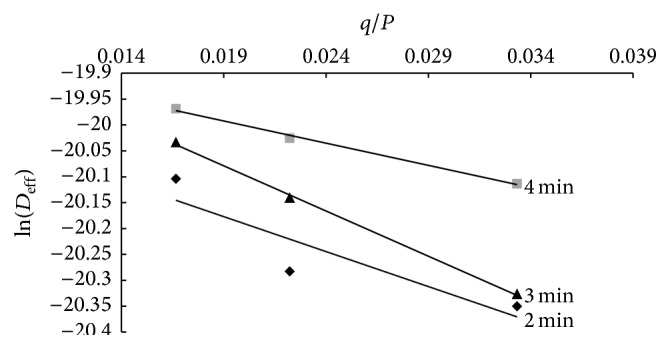
Variation of ln⁡(*D*_eff_) against *q*/*P* for the various microwave pretreatment time.

The values of the moisture diffusivity were used to estimate the activation energy for moisture diffusion, *E*_*a*_.  Figure 6 shows the variation of ln(*D*_eff_) against *q*/*P* for various pretreated mango slices dried at 70°C. The activation energy, which is the energy needed to initiate internal moisture diffusion, is an indication of the temperature sensitivity of *D*_eff_. The activation energy obtained for drying process was 13.52 W/mm for 2 min, 17.48 W/mm for 3 min, and 8.58 W/mm for 4 min pretreated samples. The activation energy values obtained in this study were lower than the 46.91 W/mm reported for microwave-vacuum-drying of tomato slices [36] and 21.6 W/g for microwave-drying of mango ginger [37] but generally higher than the 5.54 W/mm for okra [25]. The values were however within activation energy values of 13.6 W/mm and 14.945 W/mm reported for* Pandanus* leaves [28] and potatoes [34], respectively.

### 3.3. Modelling of the Drying Curves

The dimensionless moisture ratio against drying time for the experimental data at various pretreatment times and air temperatures was fitted to the Page, Henderson and Pabis, Logarithmic, and Midilli et al. models available in the literature. The results of such a fitting of the experimental data for microwave-assisted drying at 70°C are displayed in Tables 4, 5, and 6, which show the values of the estimated constants with their corresponding statistical *R* ^2^, RMSE, and *χ* ^2^ values characterizing each fitting. From the results obtained, it is evident that the experimental data fitted adequately to the models used in this study. The correlation coefficients obtained are in the range of 0.995–0.9972. This means that the three models could satisfactorily describe the microwave-assisted convective air-drying of mango slices. The relatively high values of correlation coefficients, low root mean square errors, and low reduced chi-square indicate a good predicting capacity for the temperature tested over the entire duration of the drying process. Among the models examined, the Midilli et al. model was observed to be the most appropriate one for all the experimental data with the highest value for the coefficient of determination (*R* ^2^) and lowest reduced chi-square (*χ* ^2^) and RMSE. The estimated parameters and statistical analysis of the models examined for the microwave powers at different times are illustrated in Tables 4, 5, and 6. It was observed that the value of drying rate constant (*k*) for all the models tested increased with microwave pretreatment time. This implies that drying rate potential increased with increase in microwave pretreatment time. Murthy and Manohar [37] found the Midilli et al. model to best explain the thin layer drying behaviour of stone apple slices pretreated with microwave prior to hot air-drying at different temperatures (40–70°C) in a forced convection dryer. Our findings are consistent with microwave-assisted drying of yam cubes [14].

### 3.4. Effect of Microwave Power and Pretreatment Time on Ascorbic Acid

**Figure 7:**
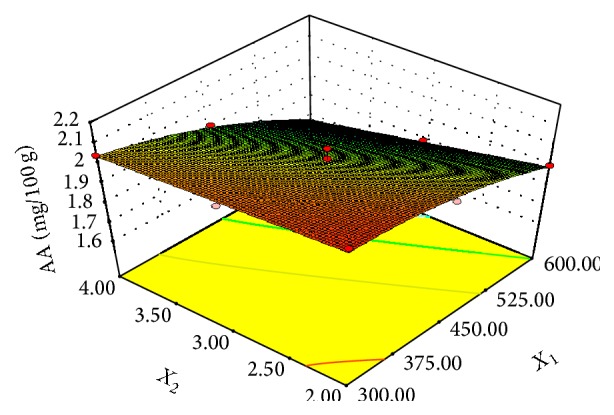
Effect of microwave power (*X*_1_) and pretreatment time (*X*_2_) on AA.

The effect of microwave power and pretreatment time on ascorbic acid (AA) of mangoes dried at 70°C is shown in Figure 7. Both power and time were significant model terms on the ascorbic acid (AA) content (Table 7). Increment in microwave power and longer pretreatment time resulted in more reduction of the AA content. The AA content of the fresh mango decreased from 3.84 mg/100 g dry matter to 1.794 mg/100 g dry matter after drying the various pretreated samples, representing 53% loss in vitamin C. In comparison with the control (2.204 mg/100 g), the AA content of the respective microwave pretreated sample was lower (2.056, 1.983, and 1.794) for 300 W, 450 W, and 600 W powers, respectively. The power of the microwave had about twice negative effect on AA degradation compared to the treatment time (Table 7). The reduction of ascorbic acid content observed for the microwave pretreated samples may be due to the destruction of vitamin C by the electromagnetic waves. The thermal effect of the microwave power coupled with the irreversible oxidative reactions due to longer drying times during hot air-drying may have contributed to the damage of about half of the AA content.

Losses of ascorbic acid during microwave-drying have been reported [38]. The degradation of AA in this present study is in agreement with results obtained by Zheng and Lu for microwave pretreatment of different parts of green asparagus [39]. Ascorbic acid losses between 10% and 50% are reported for drying food stuff [40]. In a related study involving microwave-drying of okra fruit, AA reduction between 43% and 63% was stated [41].

### 3.5. Effect of Microwave Power and Pretreatment Time on Nonenzymatic Browning

**Figure 8:**
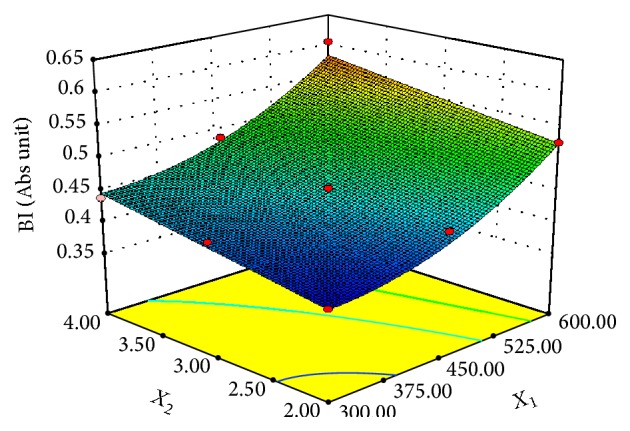
Effect of microwave power (*X*_1_) and pretreatment time (*X*_2_) on BI.

Nonenzymatic browning is another quality indicator in drying. Whereas browning is desirable in some processing food, excessive browning is undesirable in dried mangoes. The effect of microwave power and pretreatment time on nonenzymatic browning is clear (Figure 8). Brown pigment formation significantly increased with both microwave power and pretreatment time from 0.39 to 0.60 at the various condition studied. As expected, microwave power had a negative profound effect compared to pretreatment time on BI (Table 8). The control however had less browning (0.29) than all the microwave-assisted dried mangoes. This agrees with a study by [42] for dried tomato quarters at 50°C to 90°C, where the author observed that brown pigment formation increased with temperature from 0.58 to 0.68. Browning index equal to 0.60 was considered critical based on visual assessment by consumers. This increasing trend of nonenzymatic browning as a result of microwave power and treatment time is attributed to the occurrence and reaction between nitrogenous constituents and reducing sugars, nitrogenous constituents and organic acids, and sugars and organic acids [42, 43].

### 3.6. Optimization of the Drying Parameters

**Figure 9:**
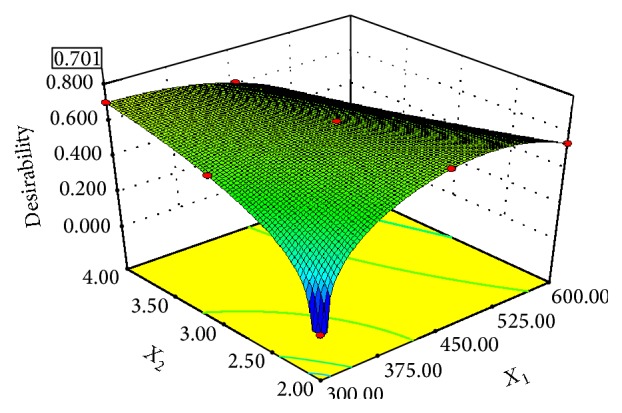
Effect of drying temperature and frying time on 3D plot of the desirability index for the optimal frying time.

The optimal microwave-assisted drying condition for mango slices was established using overall desirability index explained earlier. The maximum predicted DT, BI, and AA were 695.79 min, 0.45 Abs units, and 2.04 mg/100 g, respectively. These predicted values are closer to their corresponding experimental values of 870 min, 0.60 Abs units, and 2.13 mg/100 g. The overall desirability of 0.701 shown in Figure 9 was obtained for the microwave effect, the kinetics, and the quality of dried mango slices. In the range of the factors used, 95% confidence prediction gave optimal microwave power of 300 W and pretreatment time of 4 min. At this best condition, the DT was 695.80 min, the BI was 0.45 Abs units, and the AA was 2.04 mg/100 g dry matter.

### 3.7. Energy Consumption during Microwave-Assisted Drying

The control samples took about 990 min to dry from 4.64 g water/g dry matter to 0.04 g water/g dry matter and consumed 33 kWh of energy in the process. This value was compared with the energy consumption from the optimized microwave pretreatment conditions of 300 W/4 min to make the energy savings known as a result of microwave application. A reduction of 29.70% energy requirement was achieved because of microwave application at 300 W for 4 min. Reduction in energy consumption due to microwave application may be due to the energy efficiency advantage of microwave over the conventional method, which causes moisture migration from within rather than from the surface of the samples. Similar trends were also observed by [44] and [34] for microwave-drying of parsley and potato slices, respectively.

## 4. Conclusion

The following conclusions can be drawn from the present work where microwave-assisted air-drying of mango slices has been studied:

- Moisture migration during drying of mango slices occurs in the falling rate regime.
- Both microwave power and pretreatment time played a significant role in characterizing the drying behaviour of mango slices. Microwave power had a higher effect on drying kinetics, ascorbic acid degradation, and formation of brown pigment than pretreatment time during drying.
- Of the four empirical drying models tested in this study, the Midilli et al. model provided the best representation of mango slices. The decision was based on three statistical parameters adopted to evaluate the goodness of fit for each model.
- Microwave power of 300 W and pretreatment time of 4 min were found to be the ideal conditioning prior to convective air-drying at 70°C. This condition reduced the energy requirement for air-drying of mango slices by approximately 30%.

## Figures and Tables

**Table 1:** Mathematical models that were applied to the experimental data.

Model name	Model expression	Reference
Page	MR = exp⁡(−*kt* ^*n*^)	[15]
Henderson and Pabis	MR = *a*exp⁡(−*kt*)	[16]
Logarithmic	MR = *a*exp⁡(−*kt*) + *c*	[17]
Midilli et al.	MR = *a*exp⁡(−*kt* ^*n*^) + *bt*	[18]

**Table 2:** Three-level factorial design for two factors and results of DT, BI, and AA.

MP (W)	MT (min)	DT (min)	BI (Abs)	AA (mg/g)
450	3	690	0.45265	1.98316
300	4	690	0.4375	2.04337
300	3	810	0.42425	2.05638
600	3	570	0.5293	1.793878
300	2	870	0.3875	2.13061
450	2	750	0.44065	2.028827
600	2	630	0.52375	1.904337
600	4	570	0.6033	1.660969
450	4	630	0.48565	1.91352
450	3	730	0.4518	1.933673
450	3	730	0.4304	2.032653
Control		990	0.28645	2.203570

MP is microwave power; MT is microwave pretreatment time.

**Table 3:** Analysis of variance (ANOVA) for the effects of microwave power and time on drying time.

Source	Coefficient estimates	Sum of squares	Degree of freedom	Mean square	*F* value	*P* value, Pro > *F*
Intercept	711.05	—	1	—	—	—
Model	—	86302.39	5	17260.48	31.78	0.0009^*∗*^
*X*_1_	−100.00	60000.00	1	60000.00	110.47	0.0001^*∗*^
*X*_2_	−60.00	21600.00	1	21600.00	39.77	0.0015^*∗*^
*X*_1_ *X*_2_	30.00	3600.00	1	3600.00	6.63	0.0498^*∗*^
*X*_1_^2^	−12.63	404.21	1	404.21	0.74	0.4278^*∗∗*^
*X*_2_^2^	−12.63	404.21	1	404.21	0.74	0.4278^*∗∗*^
Lack of fit	—	1649.12	3	549.71	1.03	0.5268^*∗∗*^
*R* ^2^	0.9695	—	—	—	—	—

^*∗*^Significant (<0.0500); ^*∗∗*^not significant. Lack of fit is not significant at *P* value >0.0500.

**Table 4:** Parameters and statistical results for the various drying models for microwave pretreated samples at 300 W.

Model name	PT	Model constants	*R* ^2^	RMSE	*χ* ^2^
Page	2	*k* = 0.006, *n *= 1.031	0.9971	0.0167	0.0003
	3	*k* = 0.011, *n* = 0.947	0.9946	0.0217	0.0005
	4	*k* = 0.014, *n* = 0.928	0.9940	0.0231	0.0006

Henderson and Pabis	2	*k* = 0.007, *a* = 0.994	0.997	0.0183	0.0004
	3	*k* = 0.008, *a* = 0.973	0.995	0.0217	0.0005
	4	*k* = 0.009, *a* = 0.970	0.994	0.0231	0.0006

Logarithmic	2	*k* = 0.007, *a* = 1.000, and *c* = −0.010	0.9971	0.0167	0.0003
	3	*k* = 0.008, *a* = 0.974, and *c* = −0.002	0.9946	0.0217	0.0005
	4	*k* = 0.009, *a* = 0.973, and *c* = −0.005	0.9940	0.0231	0.0006

Midilli et al.	2	*k* = 0.005, *n* = 1.056, *a* = 0.979, and *b* = −4.2 × 10^−6^	0.9971	0.0167	0.0003
	3	*k* = 0.010, *n* = 0.963, *a* = 0.981, and *b* = −5.76 × 10^−6^	0.9953	0.0203	0.0005
	4	*k* = 0.013, *n* = 0.926, *a* = 0.984, and *b* = −17.96 × 10^−6^	0.9947	0.0216	0.0006

**Table 5:** Parameters and statistical results for the various drying models for microwave pretreated samples at 450 W.

Model name	PT	Model constants	*R* ^2^	RMSE	*χ* ^2^
Page	2	*k* = 0.003, *n* = 1.095	0.996	0.0209	0.0005
	3	*k* = 0.008, *n* = 0.960	0.990	0.0306	0.0011
	4	*k* = 0.016, *n* = 0.868	0.981	0.0405	0.0019

Henderson and Pabis	2	*k* = 0.006, *a* = 1.009	0.994	0.0250	0.0007
	3	*k* = 0.007, *a* = 0.967	0.991	0.0294	0.0010
	4	*k* = 0.008, *a* = 0.938	0.981	0.0414	0.0020

Logarithmic	2	*k* = 0.005, *a* = 1.036, and *c* = −0.042	0.997	0.0177	0.0004
	3	*k* = 0.006, *a* = 0.985, and *c* = −0.029	0.993	0.0258	0.0008
	4	*k* = 0.007, *a* = 0.951, and *c* = −0.021	0.982	0.0396	0.0018

Midilli et al.	2	*k* = 0.003, *n* = 1.106, *a* = 0.975, and *b* = −2.237 × 10^−5^	0.9972	0.01715	0.0003
	3	*k* = 0.010, *n* = 0.917, *a* = 0.977, and *b* = −6.329 × 10^−5^	0.9938	0.02449	0.0008
	4	*k* = 0.019, *n* = 0.820, *a* = 0.977, and *b* = −6.734 × 10^−5^	0.9865	0.03464	0.0016

**Table 6:** Parameters and statistical results for the various drying models for microwave pretreated samples at 600 W.

Model name	PT	Model constants	*R* ^2^	RMSE	*χ* ^2^
Page	2	*k* = 0.015, *n* = 0.865	0.988	0.0327	0.0013
	3	*k* = 0.039, *n* = 0.717	0.985	0.0340	0.0014
	4	*k* = 0.109, *n* = 0.576	0.993	0.0215	0.0005

Henderson and Pabis	2	*k* = 0.007, *a* = 0.940	0.987	0.0348	0.0014
	3	*k* = 0.009, *a *= 0.904	0.969	0.0504	0.0030
	4	*k* = 0.016, *a* = 0.919	0.949	0.0601	0.0042

Logarithmic	2	*k* = 0.007, *a* = 0.949, and *c* = −0.015	0.987	0.0338	0.0013
	3	*k* = 0.009, *a* = 0.898, and *c* = −0.011	0.969	0.0496	0.0008
	4	*k* = 0.018, *a* = 0.904, and *c* = −0.035	0.956	0.0562	0.0018

Midilli et al.	2	*k* = 0.021, *n* = 0.780, *a* = 0.984, and *b* = 7.6 × 10^−6^	0.99202	0.02672	0.0010
	3	*k* = 0.066, *n* = 0.585, *a* = 0.994, and *b* = −8.19 × 10^−6^	0.9933	0.023204	0.00078
	4	*k* = 0.15, *n* = 0.493, *a* = 0.999, and *b* = −9.11 × 10^−5^	0.9968	0.01519	0.00033

**Table 7:** Analysis of variance (ANOVA) for the effects of microwave power and microwave time on AA.

Source	Coefficient estimates	Sum of squares	Degree of freedom	Mean square	*F* value	*P* value, Pro > *F*
Intercept	1.98	—	1	—	—	—
Model	—	0.17	5	0.034	25.97	0.0014^*∗*^
*X*_1_	−0.15	0.13	1	0.13	95.65	0.0002^*∗*^
*X*_2_	−0.074	0.033	1	0.033	25.06	0.0041^*∗*^
*X*_1_ *X*_2_	−0.039	0.006094	1	0.006094	4.61	0.0846^*∗∗*^
*X*_1_^2^	−0.047	0.005506	1	0.005506	4.16	0.0968^*∗∗*^
*X*_2_^2^	−0.0005774	0.0000008	1	0.0000008	0.000638	0.9808^*∗∗*^
Lack of fit	—	0.001714	3	0.00005712	0.23	0.6224^*∗∗*^
*R* ^2^	0.9287	—	—	—	—	—

^*∗*^Significant (<0.0500); ^*∗∗*^not significant. Lack of fit is not significant at *P* value >0.0500.

**Table 8:** Analysis of variance (ANOVA) for the effects of microwave power and microwave time on BI.

Source	Coefficient estimates	Sum of squares	Degree of freedom	Mean square	*F* value	*P* value, Pro > *F*
Intercept	0.45	—	1			
Model	—	0.035	3	0.012	42.76	0.0001^*∗*^
*X*_1_	0.068	0.028	1	0.028	99.82	0.0001^*∗*^
*X*_2_	0.029	0.005078	1	0.005078	18.35	0.0036^*∗*^
*X*_1_ *X*_2_	0.007387	—	1	0.0002336	—	—
*X*_1_^2^	0.032	0.002799	1	0.002799	10.12	0.0155^*∗*^
*X*_2_^2^	0.015	—	1	0.0002767	—	—
Lack of fit	—	0.001619	5	0.0003238	2.04	0.3612^*∗∗*^
*R* ^2^	0.9483					

^*∗*^Significant (<0.0500); ^*∗∗*^not significant. Lack of fit is not significant at *P* value >0.0500.
